# Improvement in Sonographic Vasospasm Following Intravenous Milrinone in a Subarachnoid Hemorrhage Patient with Normal Cardiac Function

**DOI:** 10.7759/cureus.2916

**Published:** 2018-07-03

**Authors:** Nakul Katyal, Pravin George, Premkumar Nattanamai, Larry N Raber, Jonathan M Beary, Christopher R Newey

**Affiliations:** 1 Neurology, University of Missouri, Columbia, USA; 2 Neurology, Cleveland Clinic Ohio, Cleveland, USA; 3 Neurology, Cleveland Clinic, Cleveland, USA; 4 Neurobehavioral Sciences, A. T. Still University, Kirksville, USA; 5 Neurology, Cleveland Clinic Ohio, Akron, USA

**Keywords:** milrinone, vasospasm, subarachnoid hemorrhage, delayed ischemic neurologic deficit, transcranial doppler

## Abstract

Cerebral vasospasm and delayed cerebral ischemia are well-known complications of an aneurysmal subarachnoid hemorrhage (aSAH), generally occurring days to weeks after hemorrhagic ictus. Management strategies for these complications are controversial and vary in efficacy. There is a growing interest in supporting the use of intravenous (IV) milrinone to manage vasospasm. A 31-year-old male presented to the hospital after being found down outside his home. Computed tomography (CT) of the head and subsequent CT angiogram revealed a Fisher Grade 4 aneurysmal subarachnoid hemorrhage (aSAH). Six hours after admission, he became hypotensive and his neurological examination declined. A repeat CT head showed a new, left frontoparietal intracerebral hemorrhage (ICH) along with increasing SAH. He was stabilized with vasopressors and underwent emergent decompressive hemicraniectomy with subsequent clipping of the aneurysm. Approximately one week later, transcranial Doppler (TCD) showed increasing mean flow velocities in the bilateral anterior and middle cerebral arteries consistent with cerebral vasospasm. He was treated with intravenous milrinone. Repeat TCD 6.5 hours after the initial TCD showed improved mean flow velocities. His cardiac function by echocardiogram assessment was normal. The decrease in TCD velocity following treatment with milrinone indicates an improvement in the cerebral vasospasm regardless of cardiac output in a patient with subarachnoid hemorrhage. This case suggests that augmenting cardiac output may not be the only mechanism for the therapeutic benefit of milrinone.

## Introduction

Cerebral vasospasm and delayed cerebral ischemia (DCI) are well-known complications of aneurysmal subarachnoid hemorrhage (aSAH). A vasospasm usually occurs between Days 4 and 10 after initial SAH in about 30% of the patients but can occur days before and up to weeks after this peak period. Vasospasm may be related to delayed cerebral ischemia, which is the leading cause of morbidity following aSAH and generally presents with signs of a focal neurological change or globally as a decreased or altered level of consciousness [1-2]. Detecting neurological decline in patients with poor baseline neurological examinations poses a clinical challenge. Monitoring such patients for DCI usually depends on identifying cerebral vasospasm via a surrogate marker [3]. A monitoring tool for cerebral vasospasm is the transcranial Doppler (TCD). Once a cerebral vasospasm is identified, therapies are considered that directly counteract vasospasm and/or improve tissue perfusion [1-3]. Recent studies have shown that the phosphodiesterase 3 inhibitor milrinone is a potent vasodilator that can be used to increase cardiac output and augment cerebral blood flow [4]. In this case report, we present a patient with aSAH and a cerebral vasospasm detected by TCD who was treated with intravenous milrinone. A repeat TCD only hours later showed an improvement in the cerebral vasospasm despite unchanged cardiac output and transthoracic echocardiography (TTE). This case highlights the potential utility of intravenous milrinone in cerebral vasospasm irrespective of cardiac function.

## Case presentation

**Figure 1:**
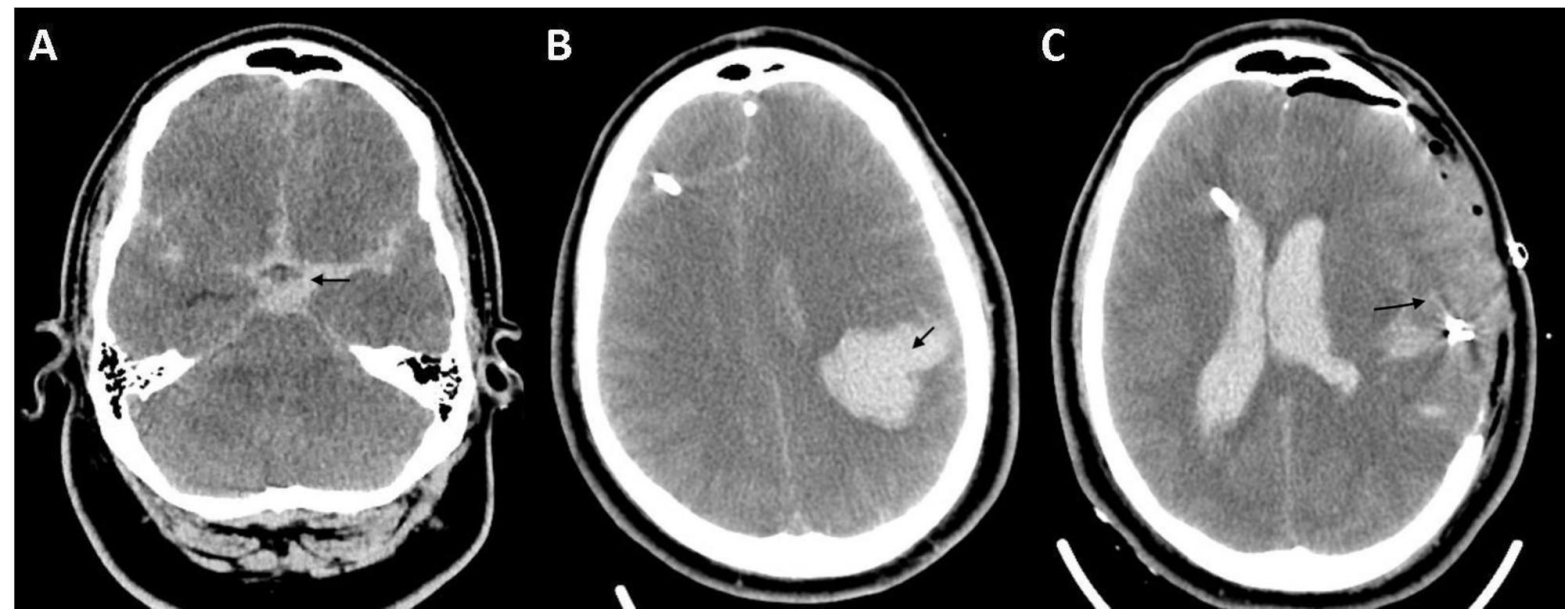
Computed tomography (CT) of the head (A) At admission: Subarachnoid hemorrhage (SAH) at the basal cisterns; (B) Six hours post-admission: left intracerebral hemorrhage was noted; (C) Two days post-decompressive craniectomy: hyperdensity in surgical bed and clips. On the left, the intraventricular hemorrhage is seen in the lateral ventricle with the external ventricular drain (EVD) in the right frontal horn.

A 31-year-old man with no known medical history presented after being found down outside his home. On arrival at the emergency department (ED), he was lethargic but following commands without extremity weakness. Mild dysarthria was noted. His blood pressure in the ED was 121/57 mmHg. He was not taking any medications and the toxicology screen was negative. Computed tomography (CT) showed SAH with a large clot burden in the basal cistern and a left Sylvian fissure (Figure 1A). There was a trace intraventricular hemorrhage (Fisher Grade 4). A CT angiography of the head and neck was obtained and was negative for a vascular abnormality/aneurysm. Serum sodium was 143 mEq/L (normal 135-145 mEq/L). Six hours after admission, he became stuporous and required intubation. Repeat CT imaging showed increased SAH and new, left frontoparietal intracerebral hemorrhage (ICH; Figure 1B). His left pupil became dilated and nonreactive. He was resuscitated with hyperventilation, hyperosmolar therapy, including mannitol and hypertonic saline, as well as sedation. An external ventricular device (EVD) was placed, which revealed an elevated opening pressure. He was hemodynamically unstable with fluctuations in blood pressure and heart rate. He was eventually stabilized with vasopressors and was taken to the operating room for emergent decompressive hemicraniectomy and clot evacuation. The distal middle cerebral arteries were subsequently clipped. The post-procedure cerebral angiography was negative for a vascular abnormality/aneurysm. CT head showed the right frontal EVD, clips in the left middle cerebral artery distribution, and decompressive hemicraniectomy (Figure 1C).

**Figure 2:**
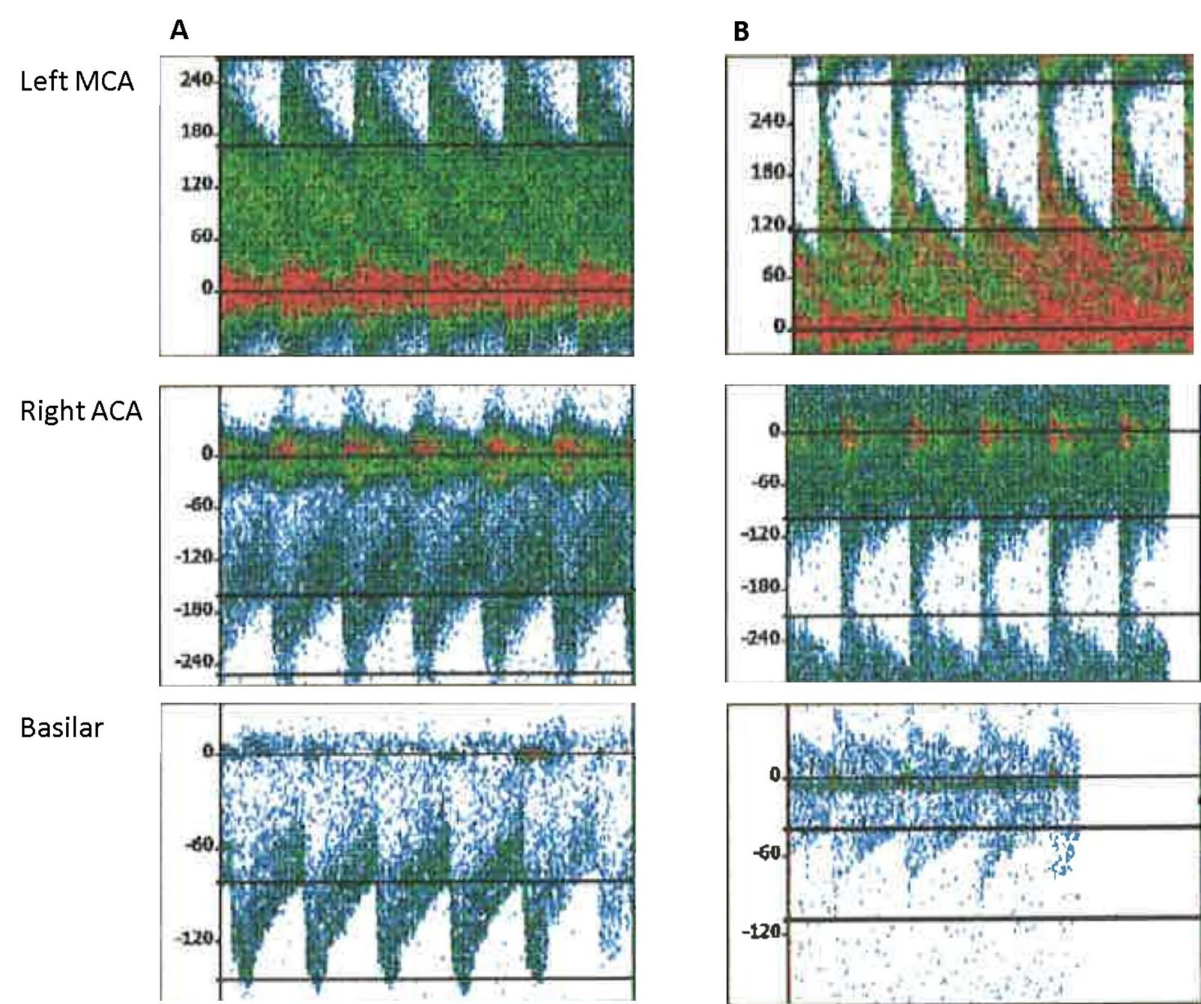
Transcranial Doppler (TCD) waveforms Transcranial Doppler (TCD) waveforms from the left middle cerebral artery, right anterior cerebral artery, and basilar artery, showing the pre-milrinone waveforms (A) and the post-milrinone waveforms (B).

Post-evacuation, he developed refractory intracranial hypertension. He continued to receive osmolar therapy and eventually required prolonged neuromuscular blockade. His serum sodium increased to 156 mEq/L. Despite the elevated ICP, his cerebral perfusion pressure was maintained. His oxygenation requirements continued to increase, requiring increasing positive end-expiratory pressure (PEEP). Chest x-ray showed increasing bilateral pulmonary opacities. He developed a refractory fever that was controlled with an advanced cooling device (Arctic Sun, Bard Medical Division, Covington, GA, US). He received broad-spectrum antibiotics for aspiration pneumonia. TTE showed a normal ejection fraction without any areas of akinesis. On Day 8 of admission, transcranial Doppler (TCD) values for the left middle cerebral artery (MCA) was 220 cm/s, right MCA was 204 cm/s, right anterior cerebral artery (ACA) was 191 cm/s, left ACA was 89 cm/s, and basilar artery was 102 cm/s. He was treated with milrinone (0.1 mg/kg bolus over 10 minutes; then 0.5-0.75 mcg/kg/min infusion). The repeat TCD on the same day at 6.5 hours after the initial TCD showed a reduction in mean flow velocities in all vascular territories. Transcranial Doppler (TCD) waveforms from the left MCA, right ACA, and basilar artery, showing the pre-milrinone waveforms and the post-milrinone waveform (Figure 2).

Left MCA velocity decreased to 171 cm/s (22.27% reduction), right MCA to 156 cm/s (23.52% reduction), right ACA to 136 cm/s (28.79% reduction), left ACA to 84cm/s (5.61% reduction), and basilar artery to 63 cm/s (38.23% reduction) as cardiac function remained largely unaffected. Mean flow velocities in different blood vessels before and after milrinone therapy (Table 1).

**Table 1:** Mean flow velocities Mean flow velocities in different blood vessels before and after milrinone therapy

Blood vessels	Mean flow velocities before milrinone administration	Mean flow velocities after milrinone administration
Left middle cerebral artery	220 cm/sec	171 cm/sec
Right middle cerebral artery	204 cm/sec	156 cm/sec
Right anterior cerebral artery	191 cm/sec	136 cm/sec
Left anterior cerebral artery	89 cm/sec	84 cm/sec
Basilar artery	102 cm/sec	63 cm/sec

His cardiac output, as monitored on FloTrac (Edwards Lifesciences, Irvine, CA, US), remained unchanged at 7.8 L/min with a stroke volume variability (SVV) of 6%. Electrocardiography (ECG) was normal and cardiac troponin testing was negative. Magnetic resonance imaging (MRI) of the brain showed the known SAH and ICH with ischemia in the left MCA territory as a result of vessel clipping. There were no areas of ischemia. Over the next few days, his neurological examination remained poor. The family ultimately withdrew care.

## Discussion

First-line therapies for managing cerebral vasospasm in patients with SAH aim at improving cerebral blood flow. Historically accepted therapies have included blood pressure augmentation and hypervolemia [5-6]. Although frequently used, these therapies are not associated with improved outcomes and can result in significant morbidity due to complications such as pulmonary edema, myocardial ischemia, hyponatremia, renal medullary washout, in-dwelling catheter-related complications, cerebral hemorrhage, and cerebral edema [5-8]. Hypervolemic therapy has the potential to reduce brain tissue oxygenation [7-8]. Induced hypertension has also been recently questioned. A randomized clinical trial evaluating the effectiveness of induced hypertension in SAH patients with delayed cerebral ischemia reported a risk ratio for poor outcome of 1.0 and a risk ratio for serious adverse events of 2.1 in patients randomized to induced hypertension as compared to those with no hypertension. This study concluded that induced hypertension may lead to significant clinical side effects [9].

National guidelines currently recommend maintaining euvolemia as a means to prevent delayed cerebral ischemia [10-11]. Augmenting cerebral blood flow by manipulating cerebral perfusion pressure is often attempted by increasing mean arterial pressure (MAP) [12]. This approach is not as effective when there is an increase in vascular resistance. Methods directly targeting vascular resistance may also be necessary. The calcium-channel antagonist nimodipine has repeatedly been shown to improve neurologic outcomes after aneurysmal SAH; however, it has not been shown to decrease radiographic vasospasm [13-14]. Other vasodilators, such as magnesium, have been studied but have not improved outcomes [15-16].

Recent studies have reported rapid neurological and angiographic improvement in cerebral vasospasm with the use of phosphodiesterase inhibitor-based vasodilators [4]. Milrinone is a phosphodiesterase 3 inhibitor that combines vasodilatory and inotropic properties by increasing adenosine 3′,5′-cyclic monophosphate or cyclic AMP (cAMP) in the cytosol of vascular smooth muscle cells and cardiomyocytes [17]. The mechanism of action of milrinone in improving cerebral vasospasm is somewhat controversial, with some authors citing augmented cerebral microcirculation and others attributing efficacy to an increase in cardiac output [18]. Studies have also reported an anti-inflammatory action in milrinone [19]. In our study, cardiac output was normal prior to initiating milrinone and remained unchanged during IV milrinone therapy, suggesting that the effect on vasospasm treatment is not secondary to cardiac output, thus supporting the microcirculation theory.

In a study assessing the efficacy of intra-arterial milrinone in 22 patients with angiographically proven cerebral vasospasm, milrinone therapy resulted in a 53 ± 37% increase in arterial diameter (P<.0001). In that study, milrinone infusion was associated with a moderately increased heart rate with systemic arterial pressure being unchanged [17]. Another study based on the review of 88 patients diagnosed with delayed ischemic neurologic deficits after SAH concluded that using IV milrinone and maintaining homeostasis require less intensive monitoring than standard hypertension, hemodilution, and hypervolemia therapy. No significant side effects or medical complications were reported with IV milrinone use. A total of 48.9 % of patients returned to their previous baseline, and 75 % of patients had good functional outcomes [4]. Our case highlighted the improvement in the TCD mean flow velocities following IV milrinone therapy.

Despite numerous studies showing the potential benefits of milrinone therapy in the reversal of cerebral vasospasm, a randomized controlled study to validate its use in vasospasm and delayed cerebral ischemia is yet to be performed. The mechanism of action of milrinone and its benefit to morbidity and mortality in this application remain unclear.

## Conclusions

Our case is one of the first to highlight sonographic improvement in vasospasm after initiating intravenous milrinone in a subarachnoid hemorrhage patient without a change in cardiac output. This suggests that the mechanism of improvement in vasospasm is not due to cardiac augmentation.
